# Building capacity for knowledge translation in occupational therapy: learning through participatory action research

**DOI:** 10.1186/s12909-016-0771-5

**Published:** 2016-10-01

**Authors:** Sally Bennett, Mary Whitehead, Sally Eames, Jennifer Fleming, Shanling Low, Elizabeth Caldwell

**Affiliations:** 1 School of Health and Rehabilitation Sciences, The University of Queensland, Therapies Annexe, Chancellors Place, Brisbane, 4072 Australia; 2 Occupational Therapy Department, Princess Alexandra Hospital (Metro South Hospital and Health Service), 199 Ipswich Road, Woolloongabba, Australia

**Keywords:** Knowledge translation, Capacity building, Theoretical Domains Framework, Barriers, Enablers, Knowledge-to-action, Participatory action research

## Abstract

**Background:**

There has been widespread acknowledgement of the need to build capacity in knowledge translation however much of the existing work focuses on building capacity amongst researchers rather than with clinicians directly. This paper’s aim is to describe a research project for developing a knowledge translation capacity building program for occupational therapy clinicians.

**Methods:**

Participatory action research methods were used to both develop and evaluate the knowledge translation capacity-building program. Participants were occupational therapists from a large metropolitan hospital in Australia. Researchers and clinicians worked together to use the action cycle of the Knowledge to Action Framework to increase use of knowledge translation itself within the department in general, within their clinical teams, and to facilitate knowledge translation becoming part of the department’s culture. Barriers and enablers to using knowledge translation were identified through a survey based on the Theoretical Domains Framework and through focus groups. Multiple interventions were used to develop a knowledge translation capacity-building program.

**Results:**

Fifty-two occupational therapists participated initially, but only 20 across the first 18 months of the project. Barriers and enablers were identified across all domains of the Theoretical Domains Framework. Interventions selected to address these barriers or facilitate enablers were categorised into ten different categories: educational outreach; teams working on clinical knowledge translation case studies; identifying time blocks for knowledge translation; mentoring; leadership strategies; communication strategies; documentation and resources to support knowledge translation; funding a knowledge translation champion one day per week; setting goals for knowledge translation; and knowledge translation reporting strategies. Use of these strategies was, and continues to be monitored. Participants continue to be actively involved in learning and shaping the knowledge translation program across the department and within their specific clinical areas.

**Conclusion:**

To build capacity for knowledge translation, it is important to involve clinicians. The action cycle of the Knowledge to Action framework is a useful guide to introduce the knowledge translation process to clinicians. It may be used to engage the department as a whole, and facilitate the learning and application of knowledge translation within specific clinical areas. Research evaluating this knowledge translation program is being conducted.

**Electronic supplementary material:**

The online version of this article (doi:10.1186/s12909-016-0771-5) contains supplementary material, which is available to authorized users.

## Background

It has been well documented that the implementation of clinical practices recommended by research is a slow and disorganised process [1], with patients often not receiving the best possible care or accessing treatments that have been proven beneficial [2]. Knowledge translation processes seek to address this problem.

Knowledge translation (KT) is defined by the Canadian Institutes of Health Research (CIHR) as “a dynamic and iterative process that includes synthesis, dissemination, exchange and ethically-sound application of knowledge” [3], with that knowledge being derived from research findings. Essentially, KT focuses on closing the gaps that exist between what is known from research evidence and that which is carried out in practice [4]. In practice, multiple factors influence the translation of research into clinical practice and the complexities of this process are widely acknowledged [5].

Responding to this complexity, there has been widespread acknowledgement of the need to build capacity and capability for KT so as to ultimately improve the quality of health care [1, 6, 7]. Research recommendations for the study of KT highlight this need explicitly, identifying the study of KT capacity building as a priority for implementation researchers, clinicians and managers [1]. Despite this need to build capacity, there is a lack of research into methods which best enable this and wide variation in the understanding of capacity building and how it is enacted in healthcare [8].

Potter and Brough [8] argued the need for a systemic approach to capacity building, proposing the presence of a hierarchy of capacity building needs. This systematic approach recognises that input into capacity building occurs at four levels: structures, systems and roles; staff and infrastructure; skills; and tools. Accordingly, building KT capacity should target not only development of staff capacity and capability, but also other levels such as organisational capacity [8]. Despite the presence of national schemes that have focused on building capacity for KT systemically [9, 10], there continues to be greater emphasis given to building capacity for KT amongst researchers [6, 11–13], with few published studies investigating the process of building KT capacity directly with clinicians. Two current approaches that have been used as methods to build KT capacity and capability amongst clinicians are secondment models [14] and training in KT skills through the use of workshops [15–18].

Gerrish and Piercy described the use of a secondment model in which 14 dieticians and nurses in clinical and academic roles from the United Kingdom were seconded to partner with teams working on KT initiatives [14]. The aim of the secondment initiative was to provide an experiential approach to KT capacity development. Focus groups with secondees and representatives from their organisations indicated that this initiative benefitted the secondees in aspects such as experiencing the KT process, and the development of various skills in KT, interpersonal relations, and change facilitation. The secondees’ organisations also benefitted from their increased clinical knowledge and professional development upon their return to their organisation. It was recognised that the benefits to the seconding organisations depend to some extent on how transferable the KT knowledge and skills are to the original practice context. It is also uncertain if such an approach is transferable to other practice contexts without the capacity to support secondment.

A second approach used in building KT capacity and capability amongst clinicians is the use of workshops and conferences to provide general training in the KT process or specific clinical areas [15–18], although limited research exists about their outcomes. For example, a workshop format including a debate and presentation of KT case studies was well received by participants in the post-workshop evaluation however subsequent application of KT within participants’ practice contexts was not assessed, and therefore the transference of these skills into their organisations is uncertain [18]. While such training opportunities are important for building KT capacity and capability, Holmes and colleagues argue that training alone is unlikely to be sufficient for increasing the uptake of research into practice, and broader approaches that consider the organisation and context are essential [19].

There is evidence that tailored KT interventions targeting known barriers to change in professional behaviour are effective when they are based on identified barriers to change [20]. We therefore undertook a project to build KT capacity within an occupational therapy department by considering the barriers and enablers to the use of KT identified by clinicians, and developing and implementing a multi-faceted KT program to support clinician’s learning and engagement with KT within the context of their clinical organisation. Further, this project aimed to contribute to a culture change within the department by facilitating KT becoming part of the norm. This paper’s aim is to describe a participatory action research project for developing this KT capacity building program for occupational therapy clinicians.

## Method

### Design

Participatory Action Research (PAR) was used to develop a KT capacity-building program for occupational therapy clinicians. According to Kemmis and McTaggart, PAR is a social, collaborative learning process where groups of individuals work together to change certain social practices [21]. A key principle of PAR is the involvement of researchers and participants working together collaboratively in a sequence of self-reflective, ongoing cycles. These cycles involve tasks such as reflecting on what might need to change (the “Reflect” phase), planning for a change (“Plan” phase), acting to bring about change (“Act” phase), observing the process and evaluation of the effects of change (the “Observe” phase), and returning again to reflection and planning and so on, in a continuous cycle. These cycles are neither rigid nor neat in reality, with cycles overlapping and plans rapidly changing in response to increased knowledge and experience. PAR seeks to facilitate participants in gaining a true development and understanding of their practices, and the circumstances in which they practice [21]. In this PAR project, researchers worked together with clinicians throughout the project to jointly respond to issues as they arose. Ethical approval for this project was granted by both hospital (HREC/12/QPAH/474) and university (2012001343) ethics committees and all participants provided their consent to participate including their consent for the study to be published.

### Participants and setting

Participants in this project included all occupational therapists working at The Princess Alexandra Hospital (PAH), a large metropolitan hospital in Brisbane Australia. Its occupational therapy department employs approximately 60 clinicians who work across a broad range of nine clinical areas: acute medical, trauma-surgical, cancer care, heart recovery, hand therapy and plastics, driving, spinal cord injury, acquired brain injury, and geriatric rehabilitation. Clinicians work in teams with a team leader and senior therapists providing leadership in each specific clinical area. Many clinicians rotate through various clinical positions in six month rotations. This department has an established, previously described [22, 23], Research and Evidence in Practice (REP) program which provides training in, and support of, evidence-based practice amongst clinicians and active involvement in research and quality improvement activities. Identification of the need to incorporate KT into this existing REP program arose through discussions with senior staff in which it was determined that while staff valued the use of evidence in clinical practice, many had difficulties implementing it and did not appear to be aware of the processes that could be used for KT. An adjustment to the REP program was therefore made to include knowledge translation processes/actions as an integral part of the existing REP program.

This project was undertaken within this single-discipline department because it was considered more manageable to use PAR processes with a group of people who were already well known to each other and had shared work practices. Further, a number of social factors including pre-existing relationships, shared governance mechanisms, and peer connection have been found to be predictive of KT success [24]. In developing capacity for KT with one health profession, it was envisaged that the participants would become more prepared and confident in interactions within their multidisciplinary teams on KT projects into the future. Further, it could serve as a pilot for the introduction of a KT program across disciplines or departments.

### Theoretical framework

The aim of the PAR project was to develop a KT program that would support clinicians’ understanding and use of the KT process generally as well as the application of this process specifically (to their teams’ clinical area) within the existing organisational context. This dual focus was seen as important for transfer of skills beyond a specific clinical area. We drew on the action cycle of the Knowledge to Action (KTA) framework [25, 26] to guide the introduction of knowledge translation to the department. The choice of this framework was based on a review of five conceptual frameworks/models that could guide KT in physiotherapy practice which concluded that the KTA framework was promising for reducing the gap between research and practice by supporting the progress and implementation of KT interventions [27].

The KTA framework is a conceptual framework involving two aspects essential to KT: knowledge creation and action. Knowledge creation is the process where knowledge is established through research or other modes of knowing such as experiential knowledge, and is distilled and synthesised into ‘knowledge products’ such as systematic reviews or clinical guidelines. The action cycle (or translation of knowledge) - developed through synthesis of planned-action research and theories [28] - then focuses on translation of these knowledge products into clinical practice. The action cycle entails: a) identifying the problem and identifying the knowledge to be translated (knowledge-to-action gaps) b) adapting the knowledge to the local situation; c) determining barriers and enablers to its use in practice; d) selecting, tailoring and using strategies to improve the use of this knowledge; e) monitoring knowledge use; f) evaluating outcomes; and g) sustaining use of knowledge over time. These seven phases of the cycle are not necessarily linear and may be iterative. The application of each phase of the action cycle within this PAR project is described below in more detail.

### Development of the KT Program: Application of the Action Cycle of the KTA framework

#### Identifying the problem and selecting the knowledge

In the initial “Reflect” phase of the PAR cycle, the problem and knowledge identification phase of the KT action cycle, was discussed with senior clinical staff, reflecting on results of a qualitative research study about the use of evidence-based practice within the department [23]. In that study, interviews were undertaken with 30 therapists in the PAH occupational therapy department to understand their perceptions of using evidence-based practice. Findings identified that clinicians were beginning to translate evidence into practice but lacked the knowledge and skills of processes for undertaking KT more effectively. Therefore a more planned and theoretically driven approach to KT was regarded as an important next step.

**Fig. 1:**
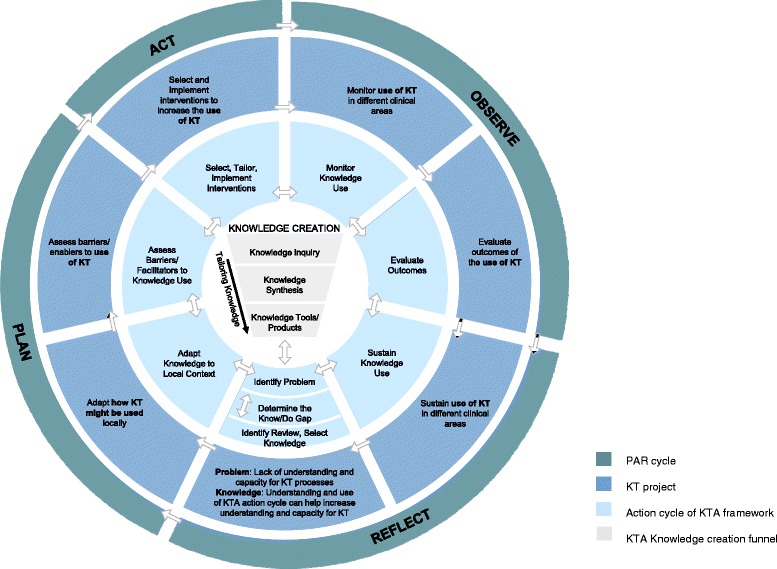
KT project phases mapped to the KTA action cycle [25] and Participatory Action Research (PAR) cycle. Modified from: Graham, Logan, Harrison, Straus, Tetroe, Caswell, Robinson, “Lost in Knowledge Translation: Time for a Map?” *The Journal of Continuing Education in the Health Professions* 26:1, 2006. [25]. Permission granted by Wolters Kluwer. Please contact the publisher for reuse terms and conditions

The identified problem in this project was the lack of understanding and capacity for KT. A meta-process was utilised in this project to translate knowledge to clinicians *about* knowledge translation. Instead of translating knowledge about a specific clinical focus (e.g. handwashing), the processes used in the action cycle described above, was considered the ‘knowledge’ to be ‘translated’. Although there is no rigorous evidence testing the use of the action cycle of the KTA framework as a process to improve KT, a number of studies have used this model to guide KT and found it to be beneficial [27, 29]. In many cases, use of PAR methods meant that the researchers and clinicians worked together to determine and carry out the processes involved in each phase of the KTA action cycle at the departmental level, in order to build their capacity for applying the action cycle themselves within specific clinical areas. In this way the PAR process helped researchers and clinicians to learn together and to ‘learn by doing’. Figure 1 shows how these processes are mapped to each other. The processes used across the first 18 months of this PAR, KT project can be seen in Additional file 1: Table S1.

#### Adapting the knowledge to the local context

Consideration was given to how the action cycle for KT could be introduced to the department as a whole as well as across different clinical areas (e.g. heart recovery, cancer care, acute medical, geriatric and rehabilitation, brain injury rehabilitation, etc) within an acute hospital setting. We planned to achieve this through involving clinicians in the various phases of the action cycle used in this project overall (e.g. identifying barriers and tailoring KT resources to suit busy clinicial practice in general), whilst at the same time supporting their use of each phase of the action cycle of the KTA framework [25, 26] to address a specific research-practice gap within their specific clinical areas (hereafter referred to as a KT clinical case study). Within these KT clinical case studies, clincians were supported in adapting the knowledge they had selected to suit their specific clincial areas.

#### Understand barriers and enablers to knowledge use:

Identification of barriers and enablers to behaviour change is not only one of the important phases within the KTA action cycle [25, 26], but it is also recognised by most other KT frameworks as essential to successful KT [2]. A survey of barriers and enablers, focus groups with clinicians, and observations by the research team, (undertaken as part of the “Plan” phase of the PAR cycle), guided our identification of barriers and enablers to the use of KT at the departmental level and subsequent selection of intervention strategies. Both the survey and observations by the research team were informed by the Theoretical Domain Framework (TDF) [30, 31].

The TDF is a validated, integrative framework of behaviour change, developed through the synthesis of behaviour change theories and key theoretical constructs. The validated version of TDF [30] contains 14 domains (knowledge; skills; social/professional role and identity; beliefs about capabilities; optimism; beliefs about consequences; reinforcement; intentions; goals; memory, attention and decision processes; environmental context and resources; social influences; emotions; and behavioural regulation) with accompanying constructs that assist in the analysis of common determinants of behaviour change. For example, in the domain of ‘beliefs about capabilities’, some constructs drawn upon are self-confidence, self-efficacy and perceived competence. It is argued that the use of the TDF allows researchers to understand barriers and enablers of behaviour change that might then inform the choice of strategies that target behaviour change [27]. The TDF is now frequently used to guide identification of barriers and enablers to specific clinical behaviours in KT projects [32–34].

No questionnaire was available at commencement of this study that asked about the use of KT processes generically that could be used across different clinical areas. We therefore designed a questionnaire to use before the KT program commenced to identify 1) participant’s use of a selection of KT behaviours (that they could think about in relation to their clinical area); 2) their perceived barriers and enablers to the use of KT processes generically; and 3) clinical and demographic variables to describe the participants. This questionnaire is known as the Knowledge Translation Questionnaire, and is available in Additional file 2: Supplementary file 2. The first section of the questionnaire included participants’ reported awareness and use of evidence from clinical guidelines and systematic reviews, reported use of barrier analysis, selection of interventions to address these barriers, and self-report of providing assessments/interventions recommended by a clinical guideline or systematic review. The second section, referred to as the ‘Barriers and enablers to the use of KT processes’, was designed to understand the key barriers and enablers that might influence the use of KT processes in general (as outlined in the KTA action cycle), and was not specific to any clinical area. It contained 38 questions mapped to the 14 domains (determinants of behaviour change) of the Theoretical Domains Framework (TDF) and its associated constructs [30, 31]. Baseline data from this section of the questionnaire was used to inform the development of KT strategies used in the KT program.

#### Select, tailor and implement interventions

Identification of barriers and enablers to the uptake of specific practices [35], can in turn inform the design of targeted interventions that aim to facilitate behaviour change amongst clinicians [36–38]. In this project the selection of interventions to address the identified barriers to the use of KT was undertaken in the first cycle of the PAR “Act” phase and repeated in the second cycle of the PAR “Act” phase by the researchers in consultation with clinical leaders. Selection of interventions was informed by research about commonly used behaviour change strategies [39], review of implementation interventions [2, 40], consideration of strategies identified by the focus groups, and the needs and resources of the local context. While particular attention was paid to matching interventions to barriers that were identified as more prominent in the survey and focus groups at the commencement of this project (for example providing education to address perceived lack of knowledge and skills in KT), interventions to boost identified enablers were also implemented (for example, clinicians identified leadership support for KT as critical, and therefore interventions to encourage leadership for KT within clinical teams was encouraged). Interventions were tailored where possible to meet the demands of the clinical context so that KT actions could be undertaken in and around regular clinical activities. Interventions were then categorised into broad types of intervention strategies. It should be noted that the PAR methodology required that the selection and implementation of intervention strategies was a shared process, iterative, and responsive to the needs of individuals and teams [41]. It involved learning together with all staff involved in the adaptation and use of KT locally.

#### Monitoring

Within the KT action cycle, monitoring is identified as a necessary step to gauge whether the knowledge is being used and behaviour change occurring, or whether changes to the planned interventions are required [25, 26]. The responses to the implementation strategies, and the use of the action cycle by each clinical team was informally discussed, monitored and evaluated by the research team, mentors, and departmental leader. Researcher’s observations of what seemed to work and what did not were documented and mapped against the domains of the TDF as field notes. Researchers tracked and recorded where each team was up to with regard their specific KT clinical case study and this was reviewed in meetings with clinicians approximately every two months. This monitoring and the next step, evaluation, were undertaken as part of the PAR “Observe” phase as can be seen in Fig. 1 and Additional file 1: Table S1.

#### Evaluate outcomes

The outcomes that are important to measure in behaviour change interventions include antecedents of behaviour change as well as actual change in behaviour (adoption) [42]. We therefore asked clinicians to complete the questionnaire again 18 months after the first survey (to measure change in use of a selection of KT behaviours, and changes in perceived barriers/enablers to the use of KT mapped to the TDF). Two further evaluative sections were added to the Knowledge Translation Questionnaire for use at follow-up. These sections asked about the perceived change in clinician’s engagement with KT and culture of KT over the past year, and the perceived usefulness of KT program strategies (at the departmental level) (See Additional file 2: Supplementary file 2). Data about changes over time will be reported on elsewhere. Additionally, we documented the number of KT case studies undertaken across the nine clinical teams, and changes in practice within these specific clinical areas. Finally, we used focus groups again, 16 months after the project commenced, to explore clinician’s experiences with using KT and to consider whether this KT program resulted in KT becoming part of the overall culture within the department. Evaluation will be undertaken again in future to understand further changes that may have occurred.

#### Sustaining use of knowledge over time

The final phase in the KT action cycle is about sustaining the changes that have been gained. We planned to assess barriers to knowledge sustainability, selecting interventions to target these barriers, and to monitor and evaluate the sustained use of knowledge translation processes and the knowledge that was translated within specific clinical areas.

## Results

Fifty-two occupational therapists participated at the beginning of this project and 42 after 18 months. However substantial staff turnover occurred during the life of this project which meant that only 20 of these therapists participated across the duration of the project. This turnover was largely due to staff taking maternity leave, secondments to other positions, recreation leave, or moving to other jobs. The vast majority of participants were female and had an approximate average of 11 years of clinical experience.

### Adapting the knowledge to the local context

Clinicians were involved in adapting the use of KT processes used in this department overall (e.g. by reviewing and revising department-specific documentation processes to support communication of KT plans and actions). At the same time, each clinical team learnt about KT by using KT processes for a specific clincal focus. This included encouraging clincal teams to think about how they might adapt the knowledge they chose to focus on within their area. For example, most teams considered how they might implement the specific knowledge within the context of having ongoing staff rotations.

#### Understanding barriers and enablers to knowledge use

The survey of barriers and enablers and the focus groups at the beginning of the project identified barriers and/or enablers in all 14 of the domains of the TDF with some being more prominent than others. The main barriers to the use of KT processes identified in the initial survey and focus groups were from the domains of ‘knowledge’ (e.g. understanding the types of KT interventions that could be used), ‘beliefs about capabilites’ (e.g. confidence in their ability to choose the best strategies to address barriers and facilitate KT), ‘environmental context and resources’ (e.g. perceived lack of time to participate in KT, and insufficient training for the use of KT) and ‘attention, memory and decision processes’ (e.g. the decision to allocate time to KT activities rather than direct clinical contact time). The focus groups also identified time pressures as a barrier with specific concern about how to manage KT case studies with staff rotating between clinical teams.

Potential enablers that were identified from the survey were that KT was seen as part of the clinician’s role and important to the department they worked in, that clinicians were optimistic about KT impacting on clincial outcomes, and that they had goals to engage in KT. The focus groups also identified a number of enablers and strategies that participants thought would be helpful including having access to mentors, encouraging leadership for KT, breaking KT activities into manageable tasks, and using journal club time for discussing KT processes. A detailed report on the barriers and enablers from the survey and results from the focus groups will be published elsewhere.

#### Selection, tailoring and implementing strategies

The reason for developing this KT program using PAR methods was not just to support clinician’s engagement with KT but also for KT to be incorporated as part of the culture of the department. Therefore it was important to address as many of these barriers as possible and support potential enablers, using strategies that ideally supported by research evidence. The KT program incorporated multiple strategies that were mapped against the TDF (see Additional file 3: Table S3). These were then categorised into ten broad intervention strategies: educational outreach; teams working on clinical case studies; identifying time blocks to allocate time to KT; mentoring; leadership strategies; communication strategies; documentation and resources to support KT; funding a KT champion one day per week; setting goals and targets for KT; and KT reporting strategies (See Additional file 1: Table S1, PAR Cycle 1 and 2: Act phases).

Educational outreach [43] was provided by two academics with knowledge and experience in knowledge translation theory and practice. This included three educational sessions at the beginning of the project (two one-hour sessions providing an introduction to KT with examples of its use in clinical settings, and a one hour session on managing change). A further one hour ‘refresher’ 12 months into the project was also provided to bring new staff up to date and act as a reminder to existing staff.

One of the key aspects of this project was that while the KT was being introduced and supported across the department as a whole, clinical teams also worked on KT clinical ‘case studies’ allowing them to ‘learn by doing’. Each of the nine clinical teams worked to identify a research-practice gap important to their clinical area that could be worked on as a team (for example screening for and assessing apraxia for people with suspected difficulties executing tasks post-stroke). Team members were then given specific tasks to carry out to help progress KT in their area and team members met on a regular basis to review progress. Working in teams meant that finding regular time to meet was important. Initially, this was largely achieved through the use of time that had been previously allocated to a monthly journal club to instead work on these team KT case studies, with the ‘commandeering’ of this time to be reviewed at the end of the project.

Support was provided to each team by three of the researchers (SB, SE, JF) who acted as formal mentors. Following the education sessions, mentors were assigned to clinical teams and met with each team for one hour between three and six times per year, to support them through each of the KT processes applied to their specific clinical area.

It is also worth highlighting the importance of leadership strategies for KT. Leadership for KT occurred at both the departmental level and team level, with shared leadership being pivotal for carrying out a research project such as this. Leaders set the tone and expectations for KT, provided support and assistance when required and addressed negative feelings about KT. Leaders shared their vision for a KT agenda across the department and understood the aim of achieving a cultural shift to enable the sustained use of KT.

Targeted communication strategies were also used by the departmental director (MW) who consciously used KT language and encouraged staff in their learning about KT on a regular basis in staff meetings. Additionally the departmental director communicated with other discipline leaders about the project encouraging their support of case study projects within multi-disciplinary teams.

A number of different resources and documentation approaches were used within the KT program and were tailored to the needs of this department. For example, a workbook that was designed to lead clinical teams through the various phases of the action cycle step by step, was developed that allowed teams to just work on one aspect of KT at a time. A summary sheet was provided so that each team’s KT case study could be captured in a one-to-two page document, to enable a quick overview and communication about where the team was up to when meeting with mentors or with new staff.

This step by step approach enabled goal setting for KT clinical case studies with specific tasks identified that could be undertaken by the team or by individual staff. Setting goals for KT was particularly important to manage the rotation of staff through different clinical areas and thus through different KT case studies. When staff moved to a new area the aim was to have clear documentation and goals with respect to the KT case study for the period of that rotation that could be discussed with newly rotated staff. The departmental director also encouraged staff to identify their goals for KT as part of their annual performance plan, so their concerns and achievements could be discussed.

The progress on these KT case studies was reported on and shared on a regular basis in meetings held with the clinical team leaders (every two months), and the teams that had seen the KT process through to implementing the identified practice, shared their progress with the department as a whole. Progress on three of these KT clinical case studies were then reported at a national occupational therapy conference: 1) assessing and providing information about apraxia for people with apraxia post-stroke 2) leisure activity maintenance program for inpatients with cancer, and 3) falls prevention for people with cognitive impairment identified in accident and emergency.

#### Monitoring

At 6 months into this PAR, KT project, while clinicians were actively trying to use the action cycle, many teams had difficulties with the process - in particular, identifying research practice gaps and undertaking barrier analysis. Teams had difficulty searching for and interpreting results from research and then understanding how they might measure relevant clinical practices. Once research gaps were identified, some teams seemed to skip the analysis of barriers and jump straight to implementing strategies to increase use of the identified clinical practice, only to find implementation to be problematic. Problem solving about these issues was undertaken. Although many barriers remained by 12 months into the project, the majority of the clinicians reported greater satisfaction and greater confidence in the use of the action cycle for KT.

At 12 months into the project, after monitoring and reflecting on the progress of the project as a whole and identification of ongoing barriers to the use of KT, funding for a KT ‘champion’ for the department was sought and prioritised. This KT champion (SE) was skilled in the use of KT and worked on a range of tasks associated with this KT program for one day per week. The KT champion provided mentoring to clinical teams, as well as mentoring individual staff as required, provided orientation to KT for new staff employed within the department, and helped continue to drive the KT program.

#### Evaluating outcomes

Evaluation of the change in the use of KT activities, perceived barriers/enablers, and overall department culture change was undertaken by repeating the survey 18 months after the initial survey (with two additional evaluative sections added); focus groups 16 months after the commencement of the project; and by recording progress made in KT clinical case studies. These results, along with details of the KT case studies undertaken, will be reported on elsewhere. Evaluation is an ongoing aspect of the project and will be undertaken again in future.

#### Sustaining use of knowledge over time

There was significant staff turnover during the first 18 months of this PAR, KT project making sustainability difficult but particularly important. However this project has achieved some initial changes in the use of KT processes, and systems to sustain this change have been considered. For example, there is an ongoing commitment to continue training for new staff; and documentation processes have been employed to help new staff or remind existing staff about continuing with the new clinical behaviours resulting from the KT clinical case studies. For example, a written summary of the specific KT focus for a particular clinical team, and what steps need to be taken next, is available for staff that rotate into that clinical team, or for newly commencing staff. Minutes of each meeting about the KT case study are also kept and made available. Refresher training is provided on an annual basis and clinicians are encouraged to present the results from their team’s KT case study at relevant conferences.

## Discussion

The multiple intervention strategies contained within this KT capacity-building program aimed to support clinicians’ understanding and application of knowledge translation within their local context and to contribute to changing the department’s culture to one in which KT is part of what is attended to on a regular basis. This PAR, KT project differs from previously published KT capacity-building interventions in several ways: 1) it focuses on clinicians, not researchers or academics; 2) it provides these clinicians with direct training, support and a structured method of undertaking the KT process (or KTA action cycle) themselves within their practice setting; 3) it targeted *all* clinicians in this department, as opposed to a minority of staff accessing secondments or courses with the intention of ‘bringing back’ the KT skills; 4) it resulted in a KT program incorporating multiple strategies such as educational outreach, mentoring, use of leadership strategies and attention to organisational and process support (as recommended by Holmes and colleagues [19]), and experiential leaning (‘learning by doing’ through case studies); and finally, 5) it used a meta-process (i.e. using the KTA framework’s action cycle to guide the uptake of the use of KT processes).

The strengths of the project include: the use of a theoretical model to guide the design, monitor and evaluate the interventions; planned evaluation of the project using both quantitative and qualitative methods; and involvement of participants in the design and refinement of a multi-faceted KT program.

Both PAR [21] and the action cycle of the KTA framework [25, 26] were useful for guiding the development of this KT capacity building program, and supported clinical teams undertaking the individual KT case studies.. Use of the KTA action cycle is evident in a growing number of studies [44–46], however the majority of existing studies addressing capacity building for KT focus on supporting researchers studying implementation or clincian-researchers, or focus on discrete clinical areas. Instead, this study used a meta process to develop capacity amongst clinicians for using KT processes across any clinical area they might work in.

Involvement of all staff in the development and implementation of this KT program was critical to its viability. Use of PAR meant that staff were consciously included in the different aspects of the research, allowing a broader understanding of barriers and enablers, and better understanding for adapting potential strategies for KT across the department than would have been identified if only the research team were involved. PAR also allows a sense of ownership of the processes,, which in itself is an important motivator for KT. Throughout the project the PAR process helped the researchers and therapists to learn together and to ‘learn by doing’.

### Limitations

We attempted to use the action cycle of the KTA framework to guide the use of KT by clinicians. We acknowledge that the lack of rigorous evidence to date establishing the use of the KTA framework itself as an effective process by which to improve KT, deviates from the accepted premise of commencing the action cycle with rigorous research for knowledge translation [2]. Thorough evaluation of this intervention using in-depth mixed methods (pre- and post-intervention staff survey and focus groups) is therefore underway and will be reported at a later date.

We believe this KT program serves as an example for other groups as to how KT capacity building might be approached. However this KT program was undertaken with a department that already had a high level of skill for evidence-based practice, had access to researcher-clinicans and academic staff with knowledge and experience in KT, and had a dedicated position for one day per week to support this program. We also acknowledge that the use of a single site and local context may limit transferability of the intervention to other settings. However plans to expand this work to other departments are currently being considered.

## Conclusions

This paper describes the development of a KT capacity-building program which aimed to support clinicians’ learning and application of knowledge translation and to address the local organisational context and culture for KT. Using PAR methods, we used the action cycle of the KTA framework not only to teach the KT process to clinicians, but also as a meta-process in which it was itself the knowledge to be translated. We believe this PAR, KT project contributes to research about knowledge translation in two ways. First, and generally, this project describes development of a multi-faceted KT program to support clinicians to learn about and use the action cycle of the KTA framework. Second, and specifically, this project provided further support that the action cycle of KTA framework can be used as a strategy to introduce and apply KT processes by both individuals and organisations.

## Additional files

Additional file 1: Table S1. KT Activities mapped to the KTA action cycle [25] and Participatory Action Research (PAR) cycle- the first 18 months (DOCX 20 kb) Additional file 2: Supplementary file 2. The Knowledge Translation Questionnaire (DOC 134 kb) Additional file 3: Table S2. Knowledge translation strategies mapped to the domains of the Theoretical Domain Framework [29] (DOCX 19 kb)

## Abbreviations

KT: Knowledge translation
KTA: Knowledge to action
PAH: Princess Alexandra Hospital
PAR: Participatory action research
REP: Research and Evidence in Practice
