# Supplementary material for: Building capacity for knowledge translation in occupational therapy: learning through participatory action research
Source: BMC Med Educ. 2016 Oct 1;16:257. doi: 10.1186/s12909-016-0771-5 (PMC5045617; doi:10.1186/s12909-016-0771-5)
Supplement: Additional file 1: Table S1. — KT Activities mapped to the KTA action cycle [25] and Participatory Action Research (PAR) cycle- the first 18 months (DOCX 20 kb) [file 12909_2016_771_MOESM1_ESM.docx]

**Supplementary file 1:** KT Activities mapped to the KTA action cycle [25] and Participatory Action Research (PAR) cycle- the first 18 months

| **Cycle** | **PAR Phase** | **Examples of KT activities mapped to the action cycle of the KTA framework** |
| --- | --- | --- |
| **Cycle 1**  (1 -12 months) | **Reflect** | ***Identify the problem*:** Clinicians valued the use of evidence in practice but had difficulties implementing it. The identified problem was that clinicians did not appear to be aware of the processes that could be used for KT.  ***Identify the knowledge*:** The ‘knowledge’ to be translated was the actual action cycle of the KTA framework. Although there is no rigorous evidence testing the use of KTA as a process to improve KT, a number of studies have used this model to guide KT and found it to be beneficial [25]**.** |
|  | **Plan** | ***Adapt the knowledge*:** There was consideration of how the action cycle could be used locally by occupational therapists across clinical areas at the hospital (e.g. heart recovery, cancer care, acute medical, geriatric and rehabilitation, brain injury rehabilitation etc).  ***Understand barriers and enablers to knowledge use*:** A questionnaire based on the Theoretical Domains Framework [29] and series of focus groups were used to understand barriers and potential enablers to using KT processes. |
|  | **Act** | ***Selection of implementation strategies to encourage the use of KT*:**  This was undertaken using two approaches: 1) by mapping the barriers identified through the questionnaire to the strategies that could address these barriers; and 2) by considering the suggestions of participants in the focus groups  ***Tailor and implement strategies*:** Categories of strategies informed by the most common barriers included:   1. **Education outreach visits**, about the various processes within the action cycle to the department as a whole (3 hours total spread over 12 months); 2. **Working in teams** on KT clinical case studies, 3. **Mentoring,** e.g. mentoring clinical leaders to define KT clinical case study topics; mentoring teams to guide their use of KT processes (3-6 times per year) 4. **Documentation** **and resources** to support KT (e.g. use of a workbook that could guide clinical teams for each step in the action cycle process, use of documentation templates to provide structure and reminders for KT (eg taking minutes of KT meetings, and summarising what has been done to date on KT clinical case studies) 5. **Identifying time blocks** to allocate time to KT, e.g. use of specific departmental meeting times or journal club time for KT 6. **KT reporting strategies** e.g. reporting on KT as part of annual performance appraisal 7. **Communication strategies** e.g. departmental lead using clear KT language, communicating about KT project to other discipline leads 8. **Leadership strategies** e.g. self-nominating leads for KT clinical case studies, providing change management training for leaders; involving clinical leads in strategy selection; departmental leader providing clear and frequent messages about the benefits of KT 9. **Setting goals/targets for KT.** This was undertaken by clinical teams and reviewed with mentors and the research team. Targets for KT were also established for the KT project as a whole. 10. The final strategy (employment of a dedicated staff member 1 day/week) was identified in the 2^nd^ PAR cycle.. |
|  | **Observe** | ***Monitoring and Evaluation of knowledge use:*** The responses to these strategies, and the use of the KT action cycle by each clinical team was informally monitored and evaluated by the research team, mentors, and departmental director throughout the life of this project. Observations and responses to these strategies were mapped against the TDF as field notes. Progress on the use of KT was mapped for each clinical team. At 6 months into the project, while clinicians were actively trying to use the KT action cycle, many teams had difficulties with the process. Although many barriers remained by 12 months into the project, the majority of the clinicians reported greater satisfaction and greater confidence in the use of the action cycle for KT. |
|  | **Reflect** | The research team met to discuss and reflect on the observations. Based on these reflections, barriers and enablers to a new set of strategies were considered to be brought to the clinicians for discussion. As part of these reflections, barriers to **sustaining** the use of KT processes were discussed.  The original **identified problem** (lack of understanding and use of KT) was discussed and the focus remained on refining the use of KT within the department. |
| **Cycle 2**  (12 -18 months) | **Plan** | The second cycle of this PAR project involved another planning phase to identify the issues that had become apparent.  ***Understanding barriers to knowledge use*:** The research team met with the leaders of the clinical teams to seek feedback on barriers to the use of the action cycle during the previous 6 months in the department overall, and within their various clinical areas.  ***Select further intervention strategies***: The team leaders confirmed the observations and reflections of the research team and further strategies that could be used to address the barriers were discussed. |
|  | **Act** | ***Implement further strategies to encourage use of KT*:**  The initial strategies were continued and enhanced through the use of the additional strategies, for example:   - Employment of a **dedicated staff member 1 day/week** to provide mentoring and support around KT in the department. (For example, to provide training in KT, assistance with documentation of KT, and reminders of other KT resources available.) - **Education** eg Refresher training for the whole occupational therapy department about the action cycle using examples from clinical teams that had commenced this process was provided; Development of a KT orientation program for new staff - Improved **documentation** to enable newly rotated staff to understand what the KT project was within their new clinical team - **KT reporting strategies** e.g. reporting on processes and outcomes of KT clinical case studies to the rest of the department and in public forums (conferences) |
|  | **Observe** | ***Monitoring and Evaluation of knowledge use:*** The responses to these strategies and the use of the action cycle by each clinical team continued to be informally monitored and evaluated by the research team, mentors, and departmental director.  At 16 months into the project, clinicians participated in another focus group about their experiences using KT processes as part of a more formal evaluation of the process. At 18 months they completed the same questionnaire that was used at baseline with additional questions evaluating their perceptions of their use of KT. |
|  | **Reflect** | The research team (including mentors and departmental director) met to reflect on the results of the questionnaire and focus groups. Based on results, a new set of strategies have been considered to help sustain this KT program and will be discussed with clinicians over the coming months. This will lead in to Cycle 3 of the PAR process where the action cycle will again be used to select, implement, monitor, evaluate and then sustain use of KT by clinical teams.  ***Sustaining knowledge use:*** As part of this reflection, the research team met with clinical team leaders to consider what would need to be done to help sustain the use of KT within this department. |

Legend:

**Bold** text in the second column refers to phases of PAR cycles

***Bold*** italicised text in the third column refers to phases of KTA action cycle
