# Supplementary material for: Building capacity for knowledge translation in occupational therapy: learning through participatory action research
Source: BMC Med Educ. 2016 Oct 1;16:257. doi: 10.1186/s12909-016-0771-5 (PMC5045617; doi:10.1186/s12909-016-0771-5)
Supplement: Additional file 2: Supplementary file 2. — The Knowledge Translation Questionnaire (DOC 134 kb) [file 12909_2016_771_MOESM2_ESM.doc]

**Supplementary file 2: The Knowledge Translation Questionnaire**

Investigators who were familiar with knowledge translation, the Knowledge to Action framework, and the Theoretical Domains Framework developed the Knowledge Translation Questionnaire and trialled it with a small group of clinicians. Minor changes were made to improve readability.

The questionnaire comprised of five sections designed specifically for this study and is available below. Sections 1 and 2 can be used at baseline and follow-up (as it was in this study). Section 3 is used only at commencement of the study. Sections 4 and 5 are designed to be used at follow-up only.

**Section 1**. This section was designed to determine the perceived use of selected KT behaviours. These behaviours were drawn from the early phases of the action cycle of the KTA framework [25] (*relevant phase indicated in italics)* and included: awareness and reading of clinical guidelines and systematic reviews (*identify, review, select knowledge*); identification and adaptation of recommended practice/s from relevant knowledge sources *(adapt knowledge to local context*); identification of barriers relevant to their practice area (*assess barriers to knowledge use*); use of strategies to overcome barrier/s; and provision of recommended practice focused on a specific assessment and/or an intervention (*select, tailor, implement interventions*). The later phases of the action cycle (*eg monitoring, evaluating, and sustaining*) will be assessed in a further stage of this project.

**Section 2 .** This section asked about the barriers and enablers to the use of KT processes in general (as outlined in the KTA action cycle). Although designed for occupational therapists, this section can be adapted to use by any health professional group. It contains 42 questions mapped to the 14 domains (determinants of behaviour change) of the Theoretical Domains Framework (TDF) and its associated constructs [30,31] (see questionnaire below). The TDF [30, 31] is a validated, integrative framework of behaviour change, developed through the synthesis of 33 behaviour change theories and 128 key theoretical constructs. The validated version of TDF [30] contains 14 domains with accompanying constructs that assist in the analysis of common determinants of behaviour change. The 14 domains include: knowledge; skills; social/professional role and identity; beliefs about capabilities; optimism; beliefs about consequences; reinforcement; intentions; goals; memory, attention and decision processes; environmental context and resources; social influences; emotions; and behavioural regulation. Each domain has a number of constructs representing components of behaviour change theories.

**Section 3** of the questionnaire gathered demographic information about the clinicians, such as the highest qualification attained, current clinical area, years of clinical experience, and years of work experience.

**Section 4** of the questionnaire was designed to understand participants’ perception of change in their engagement with KT processes at the departmental level and of the culture of KT within their department over the past year. It was only for use at follow-up.

**Section 5** of the questionnaire sought participants’ judgement about the perceived usefulness of strategies to assist with the use of KT processes over the past year. It was only for use at follow-up.

**THE KNOWLEDGE TRANSLATION QUESTIONNAIRE**

The primary purpose of knowledge translation (KT) is to address the gap between what is known from high quality research and the practical implementation of this knowledge by clinicians, to improve health outcomes and efficiencies.^25^

**Please answer the questions below.**

**Section 1: Awareness and use of evidence and KT processes**

1. Are there any clinical guidelines for your core areas of clinical practice?

**Yes/ No/ I don’t know if any are available.**

If yes, please list these clinical guidelines.…………………………………………

1. Have you read any existing clinical guidelines relevant to your clinical practice?

**Yes/ No/ None are available**

If yes, please list the clinical guidelines you have read………………..……………

1. For the clinical guidelines you have read, have you determined which recommended practices should be used in your clinical practice?

**Yes/ No**

If **yes,** please provide an example…………………………………………………

1. Are there any systematic reviews for your core clinical practice?

**Yes/ No/ I don’t know if any are available.**

If yes, please list these systematic reviews…………………………………………

1. Have you read any existing systematic reviews relevant to your clinical practice?

**Yes/ No/ None are available**

**If yes**, please list the systematic reviews you have read…………………………….

1. For the systematic reviews you have read, have you determined which recommended practices should be used in your clinical practice?

**Yes/ No**

**If yes**, please provide an example……………………………………………………..

1. During the last 3 months have you provided assessments/interventions recommended by a clinical guideline or systematic review?

**Yes I did/ No I didn’t/ None were available.**

**If yes**, please give an example………………………………………………………..

1. In the last three months, clinicians in my team have identified barriers to using recommended practices from clinical guidelines and/or systematic reviews. **Yes/ No**
2. During the last 3 months my team or I have used strategies to increase the use of these recommended practices. **Yes/ No**.

**If yes**, please tell us about a time when you implemented specific strategies to increase the use of recommended practices………………………………………..

**Section 2.** **Barriers and enablers to the use of KT processes**

Please **rate** the following items by **circling** the number that best represents your **level of agreement** with the statement. There are no right answers.

| **TDF domain and associated questions** | **Strongly Disagree** | **Disagree** | **Agree** | **Strongly Agree** |
| --- | --- | --- | --- | --- |
| **Domain: Knowledge** | | | | |
| 1. I am familiar with what constitutes high  quality, synthesised research for interventions. | 1 | 2 | 3 | 4 |
| 2. I am aware of interventions proven to be effective from high quality, synthesised research in my area of clinical practice. | 1 | 2 | 3 | 4 |
| 3. I am aware of existing research that demonstrates that knowledge translation activities are effective for increasing the use of proven interventions. | 1 | 2 | 3 | 4 |
| 4. I understand the range of strategies  available to support knowledge translation. | 1 | 2 | 3 | 4 |
| **Domain: Skills** | | | | |
| 7. I know how to identify research-practice  gaps within my clinical practice. | 1 | 2 | 3 | 4 |
| 8. I know how to identify barriers that may impact on the success of translating research evidence into my clinical practice. | 1 | 2 | 3 | 4 |
| 1. I don’t know how to use strategies to   support knowledge translation. | 1 | 2 | 3 | 4 |
| **Domain: Social/professional role and identity** | | | | |
| 1. It is important for the (occupational   therapy) profession that clinicians embrace  knowledge translation practices. | 1 | 2 | 3 | 4 |
| 1. Knowledge translation processes, (e.g.   identifying research-practice gaps) is not  an efficient use of my time as an  (occupational therapist). | 1 | 2 | 3 | 4 |
| 1. Knowledge translation is important to   (the PAH**^** Occupational Therapy department). | 1 | 2 | 3 | 4 |
| **Domain: Beliefs about capabilities** | | | | |
| 13. I am confident in my ability to identify  barriers that may impact on the successful  implementation of research evidence in my  clinical practice. | 1 | 2 | 3 | 4 |
| 14. I am confident in my ability to choose the  best strategies to address barriers to  knowledge translation. | 1 | 2 | 3 | 4 |
| 15. I don’t feel confident in my ability to  identify research-practice gaps relevant to \  my practice. | 1 | 2 | 3 | 4 |
| **Domain: Optimism** | | | | |
| 16. I don’t think learning new information  about knowledge translation will make any  difference to my clinical practice. | 1 | 2 | 3 | 4 |
| 17. I think that knowledge translation is a  positive initiative that will improve  efficiencies when using research evidence. | 1 | 2 | 3 | 4 |
| **Domain: Beliefs about consequences** | | | | |
| 18. I don’t believe that using knowledge  translation processes will result in better  outcomes for my clients. | 1 | 2 | 3 | 4 |
| 19. I would regret it if I hadn’t used  recommended practices proven to be  effective with my clients. | 1 | 2 | 3 | 4 |
| **Domain: Reinforcement** | | | | |
| 20. In my organisation there are insufficient incentives to encourage me to participate in  knowledge translation activities. | 1 | 2 | 3 | 4 |
| 21. Belonging to a department that promotes  research and knowledge translation is  motivating. | 1 | 2 | 3 | 4 |
| **Domain: Intentions** | | | | |
| 22. I am not interested in learning knowledge  translation processes or skills. | 1 | 2 | 3 | 4 |
| 23. I am still thinking about whether it is  useful to learn more about knowledge  translation. | 1 | 2 | 3 | 4 |
| 24. I intend to offer my clients proven  interventions in the next 3 months. | 1 | 2 | 3 | 4 |
| 25. I am already using knowledge translation  processes. | 1 | 2 | 3 | 4 |
| **Domain: Goals** | | | | |
| 26. I aim to develop skills for knowledge  translation activities over time. | 1 | 2 | 3 | 4 |
| 27. My goal is to be able to participate in  knowledge translation activities on a  regular basis (as time permits). | 1 | 2 | 3 | 4 |
| **Domain: Memory, attention and decision processes** | | | | |
| 28. Deciding to allocate time to knowledge translation activities, rather than direct clinical contact time is difficult. | 1 | 2 | 3 | 4 |
| 29. The REP program* reminds me to use  high quality, synthesised research in my  clinical practice. | 1 | 2 | 3 | 4 |
| **Domain: Environmental context and resources** | | | | |
| 30. There is not enough time available for me  to participate in knowledge translation  activities. | 1 | 2 | 3 | 4 |
| 31. The support my organization provides to find and access high quality, synthesised research is useful. | 1 | 2 | 3 | 4 |
| 32. The overall organisational culture of the (occupational therapy department at PAH**^**) is supportive of knowledge translation activities. | 1 | 2 | 3 | 4 |
| 33. There has been insufficient training to develop the necessary skills to employ knowledge translation activities effectively (e.g. identifying research-practice gaps, barriers to implementing research) | 1 | 2 | 3 | 4 |
| **Domain: Social influences** | | | | |
| 34. There are role models in my organisation that encourage the use of rigorous research in clinical practice. | 1 | 2 | 3 | 4 |
| 35. The beliefs of peers in my department would hinder my use of knowledge translation activities. | 1 | 2 | 3 | 4 |
| 36. The beliefs of other health professionals outside my department would hinder my use of knowledge translation activities. | 1 | 2 | 3 | 4 |
| 37. I feel unduly pressured by directions from (occupational therapy) management to participate in knowledge translation activities. | 1 | 2 | 3 | 4 |
| **Domain: Emotion** | | | | |
| 38. I am pleased to be learning and/or using knowledge translation skills and processes. | 1 | 2 | 3 | 4 |
| 39. My participation in the REP program*  makes me feel valued within my  organisation. | 1 | 2 | 3 | 4 |
| **Domain: Behavioural regulation** | | | | |
| 40. I have plans to identify and review research-practice gaps in my core areas of clinical practice. | 1 | 2 | 3 | 4 |
| 41. I have plans to identify ongoing barriers to  knowledge translation activities in my  core areas of practice. | 1 | 2 | 3 | 4 |
| 42. I have plans for using approaches to  support specific knowledge translation  activities in my core area of practice. | 1 | 2 | 3 | 4 |

***REP Program**= Research and Evidence in Practice program. This program is specific to the Princess Alexandra Hospital occupational therapy department and supports and encourages therapists’ use of evidence-based practice, knowledge translation, active involvement in research, and quality improvement activities.

**^PAH**= Princess Alexandra Hospital

**Section 3 Demographic and clinical information**

Please answer the questions that follow about yourself and your (occupational therapy) practice.

1. What is your gender?

1. Male

2. Female

2. What is your **highest occupational therapy** educational qualification? (Please circle one)

1. Diploma

2. Bachelor Degree

3. Postgraduate Coursework (Certificate / Diploma / Masters)

4. Postgraduate Research (Masters / PhD)

5. Other

3. Where did you obtain your highest occupational therapy educational qualification? (Please circle the most relevant number)

1. NSW

2. QLD

3. SA

4. WA

5. NT

6. ACT

7. TAS

8. VIC

9. Overseas

4. In terms of your employee status are you:

1. A permanent employee of QLD Health
2. Long term temporary appointee
3. Casual

5. Please indicate the number of years you have been employed in the Princess Alexandra Occupational Therapy Department.……………………………………………………….

**Section 3: Perceived change in engagement with KT and culture of KT**

| 1.My understanding of knowledge translation has increased during the last year | 1 | 2 | 3 | 4 |
| --- | --- | --- | --- | --- |
| 2.My confidence in my ability to  translate research into practice has increased during the past year | 1 | 2 | 3 | 4 |
| 3.I am more likely to seek to use practices recommended from rigorous research now than I was at the beginning of last year | 1 | 2 | 3 | 4 |
| 4.I am more aware of the recommendations from systematic reviews or randomised controlled trials in my area of practice than I was at the beginning of last year | 1 | 2 | 3 | 4 |
| 5.Knowledge translation is more ‘just part of what we do’ now than it was at the beginning of last year | 1 | 2 | 3 | 4 |

**Section 4. Usefulness of KT program strategies for developing capacity for KT**

Please rate the following strategies used to support knowledge translation (in the occupational therapy department of Princess Alexandra Hospital), in terms of how useful or helpful they have been to support you in developing capacity for knowledge translation?

|  | Not at all useful | Only a little useful | Quite useful | Very useful |
| --- | --- | --- | --- | --- |
| 1.Training sessions | 1 | 2 | 3 | 4 |
| 2. Mentoring meetings | 1 | 2 | 3 | 4 |
| 3. Using journal club time for knowledge  translation planning | 1 | 2 | 3 | 4 |
| 4. Written worksheets about different aspects  of knowledge translation | 1 | 2 | 3 | 4 |
| 5. The summary and planning sheet about our  knowledge translation plan | 1 | 2 | 3 | 4 |
| 6. Having a champion in my team to  coordinate planning for knowledge  translation | 1 | 2 | 3 | 4 |
| 7. Being reminded about potential benefits of  knowledge translation in different  departmental meetings over the last year | 1 | 2 | 3 | 4 |
| 8. Working as a team on a knowledge  translation plan | 1 | 2 | 3 | 4 |
| 9.Having a dedicated staff member to talk to  about the various activities involved in  knowledge translation | 1 | 2 | 3 | 4 |
| 10.Having departmental leadership that is  supportive of knowledge translation | 1 | 2 | 3 | 4 |
| 11.Learning about what works/doesn’t work  over time (learning by doing it) | 1 | 2 | 3 | 4 |
| 12.Other (please explain) | 1 | 2 | 3 | 4 |

**Many thanks for your time**
