# Supplementary material for: Building capacity for knowledge translation in occupational therapy: learning through participatory action research
Source: BMC Med Educ. 2016 Oct 1;16:257. doi: 10.1186/s12909-016-0771-5 (PMC5045617; doi:10.1186/s12909-016-0771-5)
Supplement: Additional file 3: Table S2. — Knowledge translation strategies mapped to the domains of the Theoretical Domain Framework [29] (DOCX 19 kb) [file 12909_2016_771_MOESM3_ESM.docx]

**Supplementary file 2**. Knowledge translation strategies mapped to the domains of the Theoretical Domain Framework [29]

| TDF Domain | Strategy |
| --- | --- |
| Knowledge | - Educational outreach: Development and provision of training for all clinical staff about KT and the processes of the action cycle at project commencement (x 2 hrs), and refresher at 12 months (following exposure to KT projects) - Orientation to KT for new staff tailored to meet novice to experienced clinician needs and link new staff to KT projects being conducted in clinical teams - Mentoring in KT for each clinical team (focusing on the team’s KT project), by staff or researchers with KT experience (Average of 3 mentoring sessions per clinical team over 12 month period) |
| Skills | - Participation of whole clinical team in specific clinical KT projects to enable skill development across the team - Mentoring in using skills from the action cycle within each team’s clinical areas - KT workbook and documentation templates developed to provide structure and drive steps for KT process completion. These were enhanced following clinician feedback about their utility - Educational outreach: Additional skill development for REP champions or leaders through change management training (x 1 hour) and regular meetings about progress of KT - Skill sharing through rotation of staff between teams. Clinical leads remained in their clinical area and orientated newly rotated staff to the KT project for that clinical area |
| Social/Professional Role & Identity | - Shared understanding of the importance of KT for PAH occupational therapy developed through key messages being delivered by leaders in departmental forums and by departmental leaders - Group norms about use of KT developed though use of drivers such as team meetings, performance plan requirements and supervision structure - Departmental leader sharing KT project and goals with other allied health disciplinary leaders within the hospital - Providing opportunities to individuals to lead KT clinical projects |
| Beliefs about Capabilities | - The team based approach to KT projects enabled more experienced staff (and mentors) to support less experienced staff in outcome achievement. - Leadership team encouraging the inclusion of performance goals in annual performance plans enabled opportunities for staff to discuss their skills in KT with supervisors and the department’s EBP/KT coordinator. - Mentors reinforced clinical team’s skills in KT and discussed further learning and support needs against specific projects. - Presentation of clinical team KT case studies to the department and at conferences to provide recognition of successes and develop confidence in presenting about KT projects |
| Optimism | - Department leaders, mentors and EBP/KT coordinator provide support and encouragement for undertaking KT and were responsive to meeting the staff’s needs for KT development - Team discussion and department presentations provided opportunities for staff to discuss their views about KT and receive peer feedback - Department leadership promoting progress in KT to other disciplines. - Feedback sought on KT initiatives from other allied health disciplines and medical colleagues. Encouraging feedback from these other health professionals has resulted in significant optimism |
| Beliefs about Consequences | - Training about the benefits of KT - Discussion with mentors about pros and cons of using KT - Leadership communication about the benefits of using KT in practice |
| Reinforcement | - Including KT goals in performance planning process to drive the need to complete KT goals - Leadership recognition of team achievements in public forums and newsletters - Discussion of KT clinical ‘case studies’ as part of professional development - Regular meetings between mentors and each clinical team - Refresher training in KT at 12 months (following exposure to KT projects) - Presentation of clinical team KT case studies to the department |
| Intentions | - Teams setting goals for KT clinical case studies at each rotation - Orientation of new staff and newly rotated staff to the teams KT goals to facilitate shared understanding and early adoption of KT project |
| Goals | - Clinical teams identifying KT goals with their team - Clinical teams setting specific KT targets about what will be achieved within time frame of the rotation - Performance plan KT goals for individuals to achieve specific outcomes or knowledge |
| Memory, Attention & Decision Processes | - Reminder by departmental leader and mentors about the importance of KT - Progress reporting by team leaders and REP champions - Reminders by team leaders at supervision meetings about performance plan goals - Documentation of KT activities in the department reporting processes that are circulated across the department |
| Environmental Context & Resources | - Development of a step by step KT workbook and other documentation strategies to support completion of KT case studies - Provision of time to work on KT case studies in each clinical area - Employment of a dedicated staff member 1 day/week to support KT activities (EBP/KT coordinator) - Engaging multidisciplinary team as needed in KT clinical case study - Mentoring support to guide selection of evidence-practice gaps to focus KT clinical case studies - Librarian staff and mentors support for locating appropriate evidence sources |
| Social Influences | - Working as a team to implement the action cycle in clinical case studies - Mentoring - Presentations on KT and change management by ‘opinion leaders’ - Leadership direction about KT provided verbally and in departmental operational and strategic plans - Allied health, Nursing and medical colleague reinforcement of use of KT |
| Emotion | - Providing multiple forums (eg mentoring meetings, focus groups, leadership meetings) for all staff to discuss difficulties and frustrations with using KT, and problem-solving |
| Behavioural Regulation | - Using clinical case studies to practice the use of KT processes - Self-monitoring use of KT in specific clinical areas - Self-monitoring of the use of KT language and processes across the department |
